# Quality of Life in Patients and Their Spouses and Cohabitating Partners in the Year Following a Cancer Biopsy (the Couples Cope Study): Protocol for a Prospective Observational Study

**DOI:** 10.2196/52361

**Published:** 2024-06-18

**Authors:** Patricia I Moreno, Sarah M Worch, Jessica L Thomas, Rebecca L Nguyen, Heidy N Medina, Frank J Penedo, Judith T Moskowitz, Betina Yanez, Sheetal M Kircher, Shilajit D Kundu, Sarah C Flury, Elaine O Cheung

**Affiliations:** 1 Department of Public Health Sciences University of Miami Miller School of Medicine Miami, FL United States; 2 Department of Psychology Lynn University Boca Raton, FL United States; 3 Department of Medical Social Sciences Northwestern University Feinberg School of Medicine Chicago, IL United States; 4 Department of Psychology University of Maryland, Baltimore County Baltimore, MD United States; 5 Departments of Medicine & Psychology University of Miami Miami, FL United States; 6 Division of Hematology and Oncology Department of Medicine Northwestern University Feinberg School of Medicine Chicago, IL United States; 7 Department of Urology Northwestern University Feinberg School of Medicine Chicago, IL United States; 8 Department of Urology Vanderbilt University School of Medicine Nashville, TN United States; 9 Hinge, Inc New York, NY United States

**Keywords:** quality of life, cancer, biopsy, couples, dyads, caregiver, patients, patient, cancer biopsy, spouse, spouses, partner, partners, diagnostic, diagnostic procedure, feasibility of recruitment, recruitment, prostate biopsy, breast biopsy, screening, electronic health record, survey, surveys

## Abstract

**Background:**

Receiving a diagnosis of cancer is a profound and often very stressful experience. Few studies have prospectively recruited patients prior to receiving a new diagnosis of cancer and included spouses or partners.

**Objective:**

The aim of the Couples Cope Study is to understand the impact of undergoing a diagnostic biopsy and receiving a new cancer diagnosis on quality of life (QoL) in both patients and their spouses or partners, as well as on the quality of their relationship. This protocol paper describes the study design and assesses the feasibility of recruitment and retention.

**Methods:**

Study staff reviewed the schedules of collaborating physicians using specific encounter codes to identify patients scheduled for breast or prostate biopsies. Potential participants were prescreened via the electronic health record and sent a recruitment letter at least 2 to 3 weeks prior to their biopsy procedure. Patients subsequently underwent a phone screening to determine eligibility. Patients who enrolled provided study staff with contact information for their spouses or partners. All consent forms were completed online. Surveys were completed online prior to receiving the biopsy results (baseline), and at 1, 3, 6, and 9 months after the biopsy. Study staff engaged in ongoing, personalized contact with participants and sent assessment completion reminders via phone and email.

**Results:**

A total of 2294 patients undergoing a breast or prostate biopsy were identified and 69% (n=1582) were eligible for phone screening following electronic health record prescreening. Of the 431 patients who underwent phone screening, 75% (n=321) were eligible to participate. Of the eligible patients, 72% (n=231) enrolled and 82% (n=190) of enrolled patients had an accompanying partner or spouse who also enrolled. A total of 77% (34/44) of patients who received a cancer diagnosis and 72% (26/36) of their spouses or partners were retained through 9 months, while 80% (53/66) of patients who received a benign diagnosis and 68% (42/62) of their partners were retained.

**Conclusions:**

Prospective recruitment of patients undergoing diagnostic biopsy and their partners is feasible and requires both strategic collaboration with providers and concerted prescreening and recruitment efforts by study staff. Importantly, this study was able to conduct all study activities online without disrupting clinical workflow and without requiring patients and their spouses or partners to come into the laboratory. Consideration should be given to the ratio of biopsies to cancer diagnoses, which can vary significantly by cancer type. Prospective studies are needed and can inform our ability to provide effective support earlier to couples facing a possible cancer diagnosis. Future studies should examine other tumor types that have received less attention in QoL studies, include behavioral and neurobiological assessments beyond self-report measures, and follow couples beyond 9 months in order to examine long-term effects on QoL.

**International Registered Report Identifier (IRRID):**

DERR1-10.2196/52361

## Introduction

### Overview

Receiving a diagnosis of cancer is a profound and often very stressful experience. A new diagnosis of cancer introduces uncertainty and distress as individuals navigate treatment decisions, experience disruptions to social or occupational roles and routines, and undergo arduous treatments such as surgery, radiation, and chemotherapy [1,2]. Individuals who are newly diagnosed with cancer commonly experience decrements in quality of life (QoL) and are at elevated risk for psychological and physical symptoms, including anxiety, depressed mood, fatigue, and pain [1,3,4]. A cancer diagnosis and its subsequent treatment significantly impact not only patients but also their spouses and romantic partners. Spouses or partners are by far the most common informal cancer caregivers, helping patients manage disease symptoms, treatment side effects, and emotional distress [5]. However, caregiving in the cancer context is associated with well-documented risks to psychological and physical health [5-13].

Despite the fact that the impact of cancer extends well beyond the patient, and that patient and caregiver well-being are closely linked and reciprocally influence each other [14-20], few studies incorporate the assessments of spouses or partners or examine how both patients and their spouses or partners report cancer has affected the quality of their relationship. Furthermore, although there is widespread interest in prospective research on QoL following a cancer diagnosis, there is a paucity of non-epidemiological studies that have prospectively recruited patients prior to receiving a new diagnosis of cancer [21-26]. A 1998 study by Northouse et al [27] recruited couples prior to a woman’s diagnostic breast biopsy and examined psychosocial adjustment to either a malignant or benign diagnosis at 60-day and 1-year follow-ups. Results demonstrated that couples who received a breast cancer diagnosis reported worse psychosocial adjustment and a greater decrease in marital functioning than couples who received a benign diagnosis. Furthermore, psychosocial adjustment was highly interrelated between women with breast cancer and their spouses. To our knowledge, since this landmark study 25 years ago, no study has prospectively examined trajectories of QoL among both patients and their spouses or partners from prior to a diagnostic biopsy through the year following a malignant or benign diagnosis.

### Couples Cope Study

The goal of the Couples Cope Study is to understand the impact of undergoing a diagnostic biopsy and receiving a new cancer diagnosis on QoL in both patients and their spouses or partners, as well as on the quality of their relationship. Using a prospective observational study design and quantitative approaches that model actor-partner effects, we will examine predictors and trajectories of QoL, individual well-being, and relationship quality and functioning among couples who undergo a cancer biopsy. Patients and their spouses or partners will be recruited prior to a breast or prostate biopsy and complete follow-up assessments for 9 months after the biopsy. Comprehensive assessments will include multiple domains of QoL, such as psychosocial adjustment, social well-being, sexual functioning, symptom burden, and physical functioning. Couples will be stratified into 2 groups based on biopsy result (ie, malignant vs benign). The aim of this study is to describe the Couples Cope Study protocol and examine the feasibility of recruitment and retention of patients undergoing diagnostic biopsy and their spouses or partners.

## Methods

### Overview of Study Design

**Figure 1:**
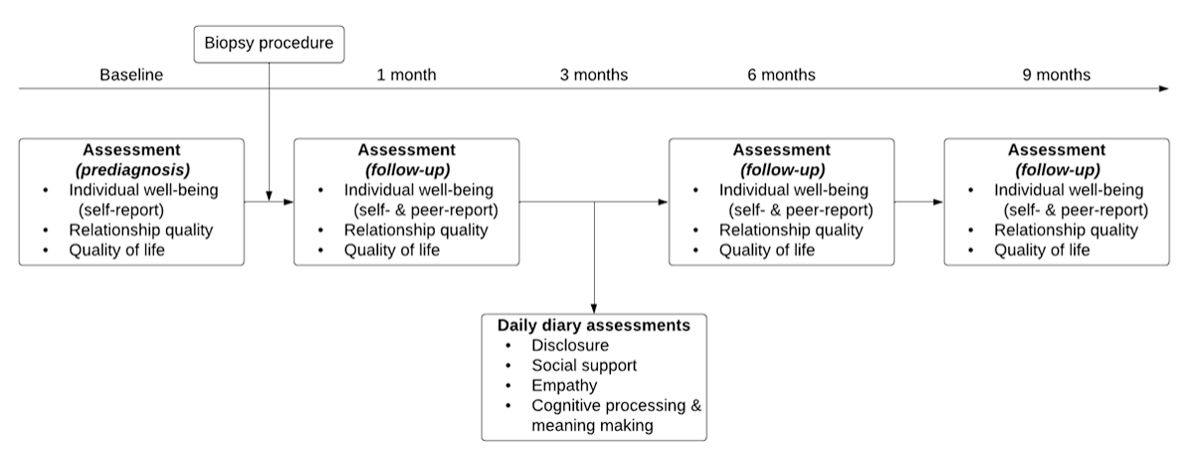
Participant flow diagram for patients and cohabitating partners or spouses; baseline and 1-, 3-, 6-, and 9-month follow-ups.

This is a prospective, nontherapeutic observational study of patients undergoing a diagnostic biopsy for prostate or breast cancer and their spouses or partners. Participants completed a baseline assessment prior to receiving the biopsy result and follow-up assessments at 1, 6, and 9 months after the biopsy, as well as daily diary assessments over a 1-week period at 3 months after the biopsy (Figure 1). Data were collected between April 2018 and November 2020. Study procedures were not altered in response to the COVID-19 pandemic as the study design included only remote contact with participants.

### Ethical Considerations

All study procedures were reviewed and approved by the institutional review board at the Northwestern University Feinberg School of Medicine (STU00206691). All participants provided informed consent prior to participation via an electronic consent form. Survey data are stored in encrypted form on Northwestern University Feinberg School of Medicine computers on its corresponding secure, password-protected server. Prior to analysis, data were deidentified and stripped of all relevant personal health information. Participants were compensated US $10 for the baseline assessment, US $20 for each follow-up assessment (3 in total), and US $5 for each daily diary assessment (7 in total), for a possible total of US $105 per participant. The payment was dispensed in the form of a prepaid Visa debit card.

### Inclusion and Exclusion Criteria

Patients and their spouses or partners were eligible if they were aged 18 years or older, married or cohabitating, able to speak and read in English, had access to the internet via at least 1 web-enabled device (eg, computer, tablet, or smartphone), and undergoing a diagnostic biopsy for prostate or breast cancer (patients only). Patients were ineligible if they had a previous cancer diagnosis (except nonmelanoma skin cancer like basal cell carcinoma), evidence of distant metastasis (stage IV cancer), inpatient psychiatric treatment in the past 6 months, and were unable to consent.

### Recruitment and Enrollment

Authorized study staff reviewed the schedules of collaborating Northwestern Medicine physicians in the Department of Urology and Lynn Sage Comprehensive Breast Center using specific encounter codes to identify patients scheduled for breast or prostate biopsies. The encounter codes used for prescreening via physicians’ electronic schedules were (1) “biopsy” for patients with prostate cancer and (2) either “US guided breast biopsy core” or “mamo stereo core ndl bx.” Potential participants were prescreened via the electronic health record (EHR) and sent a recruitment letter that introduced the study along with the study flyer at least 2 to 3 weeks prior to their biopsy procedure. Approximately 1 week after receipt of the recruitment letter, study staff called patients to provide additional information about the study and screened patients to assess their eligibility over the phone. Patients who were eligible and enrolled, provided study staff with their own contact information and that of their spouse or partner. If a patient and his, her, or their spouse or partner were determined to be eligible following phone screening, study staff sent each potential participant an email with a link to the online consent form and the baseline assessment. Study staff reviewed the consent form, including all study procedures, with potential participants on the phone in order to ensure understanding and respond to any questions or concerns. Completion of the online consent form allowed participants to proceed to the baseline assessment.

### Assessments

Baseline and follow-up study assessments, including variable domains and measures, time points, and sources of data, are described in Multimedia Appendix 1 [28-48]. All surveys were completed online. If patients received a malignant biopsy result (ie, cancer diagnosis), patients and their spouses or partners completed follow-up assessments at 1, 6, and 9 months after the biopsy, as well as daily diary assessments over a 1-week period at 3 months after the biopsy. If patients received a benign biopsy result (ie, no cancer diagnosis) and were among the first 80 couples to enroll in the study, patients and their spouses or partners completed follow-up and daily diary assessments across the same time frame. After the first 80 couples, patients who received a benign biopsy result and their spouses or partners completed only the baseline assessment.

The baseline assessment captured medical and sociodemographic characteristics (eg, age, sex, gender identity, marital status, parental status, race, ethnicity, relationship length, education, and income). EHRs were also reviewed by study staff in order to capture diagnostic biopsy results and, if applicable, cancer stage and cancer treatments (eg, surgical resection, radiation, chemotherapy, and hormone therapy). Both baseline and follow-up assessments included measures of individual well-being (ie, gratitude, empathy, meaning and purpose, attachment style, relatedness, and social support), relationship quality (ie, relationship quality and social constraints), and QoL (ie, depressive symptoms, positive and negative affect, life satisfaction, stressful life events, financial toxicity, and sexual functioning). Follow-up questionnaires also included peer reports of specific measures of individual well-being (ie, participants reported their perception of their spouses’ or partners’ gratitude, empathy, and meaning and purpose). Cancer-specific measures of health-related QoL and symptom burden, intrusive thoughts and avoidance, and supportive care needs were included in follow-up questionnaires only for patients who received a cancer diagnosis. The baseline and follow-up questionnaire batteries were expected to take approximately 30 and 50 minutes to complete, respectively.

Daily diaries assessed dyadic processes (ie, disclosure, social support [general, emotional, informational, instrumental, and cancer-specific], empathy, and cognitive processing and meaning-making) using items adapted from previous studies [49-51]. Daily diary assessments, including variable measures and response options, are described in Table 1. Daily diaries were expected to take 5 minutes or less to complete.

**Table 1:** Daily diary assessments at 3-month follow-up for patients and cohabitating partners or spouses.

Variable and measure		Response options	Respondents
**Disclosure**			
	“Did you discuss stressful or negative events with your romantic partner today?”	Yes or no	All participants
	(If yes) “Was it about your cancer diagnosis/treatment?”	Yes or no	Patients with cancer
	(If yes) “Was it about your experience caregiving or your partner’s cancer diagnosis?”	Yes or no	Spouses or partners of patients with cancer
**Social support**			
	“How much support did your partner provide today?”	0=none, 1=a little, 2=some, 3=quite a bit, and 4=a lot	All participants
	“How much comfort/reassurance did your partner provide?” (emotional support)	0=none, 1=a little, 2=some, 3=quite a bit, and 4=a lot	All participants
	“How much advice/information did your partner provide?” (informational support)	0=none, 1=a little, 2=some, 3=quite a bit, and 4=a lot	All participants
	“How much help/assistance did your partner provide?” (instrumental support)	0=none, 1=a little, 2=some, 3=quite a bit, and 4=a lot	All participants
	“How much did your partner help you cope with your cancer experience or other stressful events today?” (cancer-specific support)	0=none, 1=a little, 2=some, 3=quite a bit, and 4=a lot	Patients with cancer and their spouses or partners
**Empathy**			
	“How empathic or caring was your partner toward you today?”	0=not at all, 1=a little bit, 2=moderately, 3=quite a bit, and 4=extremely	All participants
**Cognitive processing and meaning making**			
	“To what extent did you actively reflect on your cancer experience or stressors today?”	0=not at all, 1=a little bit, 2=moderately, 3=quite a bit, and 4=extremely	Patients with cancer
	“To what extent did you try to understand or make sense of your cancer experience or stressors today?”	0=not at all, 1=a little bit, 2=moderately, 3=quite a bit, and 4=extremely	Patients with cancer
	“To what extent did your cancer experience or stressors make you think about what is important and the meaning in your life today?”	0=not at all, 1=a little bit, 2=moderately, 3=quite a bit, and 4=extremely	Patients with cancer
	“To what extent did you actively reflect on your cancer experience or stressors today?”	0=not at all, 1=a little bit, 2=moderately, 3=quite a bit, and 4=extremely	Spouses or partners of patients with cancer
	“To what extent did you try to understand or make sense of your cancer experience or stressors today?”	0=not at all, 1=a little bit, 2=moderately, 3=quite a bit, and 4=extremely	Spouses or partners of patients with cancer
	“To what extent did your cancer experience or stressors make you think about what is important and the meaning in your life today?”	0=not at all, 1=a little bit, 2=moderately, 3=quite a bit, and 4=extremely	Spouses or partners of patients with cancer
	“To what extent did you actively reflect on your stressors today?”	0=not at all, 1=a little bit, 2=moderately, 3=quite a bit, and 4=extremely	Benign biopsy patients and their spouses or partners
	“To what extent did you try to understand or make sense of your stressors today?”	0=not at all, 1=a little bit, 2=moderately, 3=quite a bit, and 4=extremely	Benign biopsy patients and their spouses or partners
	“To what extent did your stressors make you think about what is important and the meaning in your life today?”	0=not at all, 1=a little bit, 2=moderately, 3=quite a bit, and 4=extremely	Benign biopsy patients and their spouses or partners

### Retention Strategies

Strategies to optimize engagement and retention included reminder calls, text messages, and emails prior to upcoming assessments (tailored according to participants’ preferences) and providing participants with study contact phone numbers and emails to facilitate communication and accessibility. Flexible questionnaire administration online also eliminated the need for participants to travel to in-person assessments. In addition, our study team sent holiday cards that were personally signed by the principal investigators and lead study coordinator.

## Results

**Figure 2:**
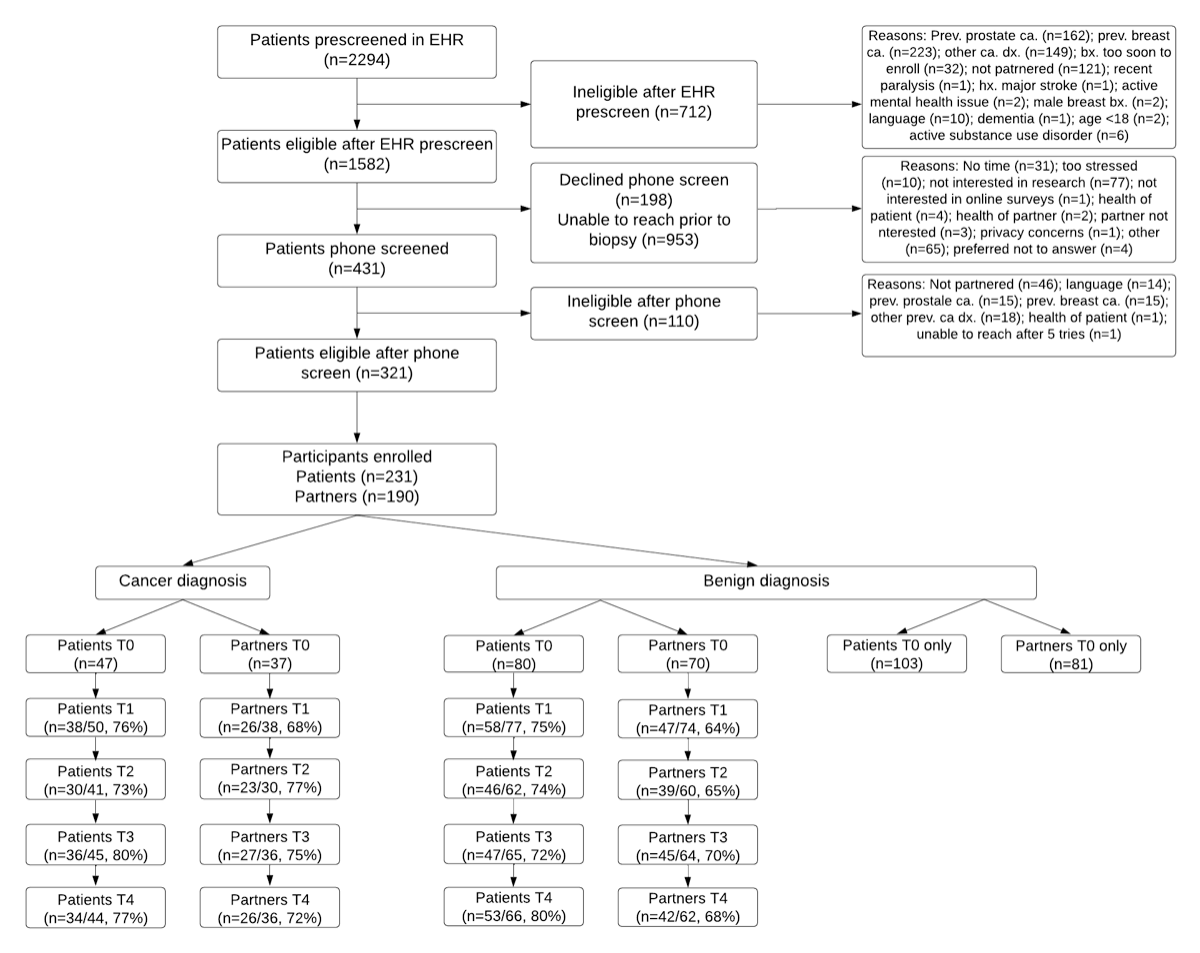
CONSORT diagram for Couples Cope Study protocol. A prospective, nontherapeutic observational study of patients undergoing a diagnostic biopsy for prostate or breast cancer and their spouses or partners. bx: biopsy; ca.: cancer; CONSORT: Consolidated Standards of Reporting Trials; dx: diagnosis; EHR: electronic health record; hx: history; prev.: previous.

As seen in the Consolidated Standards of Reporting Trials (CONSORT) diagram (Figure 2), 2294 patients undergoing a breast or prostate biopsy were identified and prescreened in the EHR. Of those prescreened in the EHR, 69% (n=1582) were eligible for subsequent phone screening and 31% (n=712) were ineligible. Common reasons for patient ineligibility in EHR prescreening included a previous diagnosis of prostate (n=162), breast (n=223), or other (n=149) cancer or not being partnered (n=121). Of the 1582 patients eligible for phone screening, 27% (n=431) underwent phone screening, 13% (n=198) declined phone screening, and 60% (n=953) were unable to be reached. Common reasons for declining phone screening included not being interested in research (n=77), not having time (n=31), and feeling too stressed (n=10). Of the 431 patients who underwent phone screening, 75% (n=321) were eligible to participate and 25% (n=110) were ineligible. Common reasons for patient ineligibility in phone screening included a previous diagnosis of prostate (n=15), breast (n=15), or other (n=18) cancer or not being partnered (n=46). Of the 321 eligible patients, 72% (n=231) enrolled and 82% (n=190) of the enrolled patients had an accompanying spouse or partner who also enrolled.

On average, patients who enrolled were 50.4 years old (SD 12.5; min: 20, max: 80) and their spouses or partners were 50.8 years old (SD 11.8; min: 21, max: 85). Patients were primarily non-Hispanic White (81%) with some representation of Asian (9%), Black (7%), multiracial (2%), and Native American (<1%) patients. Additionally, 7% of patients were Hispanic. Similarly, spouses or partners were primarily non-Hispanic White (78%) with some representation of Black (8%), Asian (7%), multiracial (4%), and Native American (<1%) spouses or partners. Additionally, 6% of spouses or partners were Hispanic. Approximately 2% of patients and 4% of spouses or partners identified as sexual or gender minorities. More patients underwent breast biopsies (73%) than prostate biopsies (27%). However, 46% of those undergoing a prostate biopsy received a cancer diagnosis, while only 12% of those undergoing a breast biopsy received a cancer diagnosis. A total of 77% (34/44) of patients who received a cancer diagnosis and 72% (26/36) of their spouses or partners were retained throughout the 9-month follow-up assessment, and 80% (53/66) of patients who received a benign diagnosis and 68% (42/62) of their spouses or partners were retained through the 9-month follow-up assessment.

## Discussion

### Principal Findings

The goal of the Couples Cope Study is to understand the impact of undergoing a diagnostic biopsy and receiving a new cancer diagnosis on QoL in both patients and their spouses or partners, as well as on the quality of their relationship. Our preliminary data demonstrate that prospective recruitment of patients undergoing diagnostic biopsy and their spouses or partners is feasible and requires both strategic collaboration with medical providers and concerted prescreening and recruitment efforts by study staff. The majority of patients who underwent phone screening were eligible to participate (72%) and most patients who were eligible to participate actually enrolled (75%). Furthermore, most patients who enrolled had an accompanying spouse or partner who also enrolled (82%). Retention through the study duration was also generally good and was higher among patients with either a malignant or benign biopsy result (77%-80%) and spouses or partners of patients with cancer (72%) than spouses or partners of patients with a benign biopsy (68%).

Importantly, the design of the Couples Cope Study allowed us to conduct all study activities remotely (either via phone or online) without disrupting clinical workflow and without requiring patients and their spouses or partners to come into the laboratory. Previous studies that have prospectively recruited patients prior to receiving a new diagnosis of cancer have primarily relied on in-clinic recruitment by physicians or medical or research staff [22-26]. These approaches are resource-intensive and can disrupt clinical workflows during sensitive medical appointments. In fact, patients with cancer are more likely to enroll in a research study when they are contacted at home [52]. Given that these factors of convenience influence enrollment, online recruitment and participation in prospective studies may be desirable alternatives to in-person approaches with people faced with a possible cancer diagnosis.

The most resource-intensive aspect of recruitment for this study was the EHR prescreening and outreach for phone screening. Even though most patients were eligible for phone screening after the EHR prescreening (69%) and only a minority of patients actively declined phone screening (13%), stress and the short time frame between scheduling a diagnostic biopsy and the completion of the procedure most likely contributed to our inability to reach a large portion of patients who were eligible for phone screening prior to their biopsy (60%). Furthermore, consideration should be given to the ratio of biopsies to cancer diagnoses, which can vary significantly by cancer type. In our sample, almost half of the patients undergoing a prostate biopsy received a cancer diagnosis, while approximately 1 in 8 patients undergoing a breast biopsy received a cancer diagnosis.

### Strengths

The strengths of this study include comprehensive psychosocial assessments that incorporate measures of both distress and symptom burden, as well as protective factors like positive affect and social support. An additional strength is the use of multimodal measurement, including daily diary sampling to examine dyadic processes as predictors or mechanisms of QoL and peer-reported measures of individual well-being to examine observability and convergent validity. Our assessment of relationship quality in both patients and their spouses or partners will allow us to capture each member’s perspective and examine correspondence in evaluations of relationship quality. Correspondence (or lack thereof) may provide a unique indicator of relationship dynamics, which may also predict QoL. A prospective design that includes a baseline assessment prior to biopsy allows us to predict QoL based on individual and relational protective and risk factors present prior to a cancer diagnosis and reduce the recall bias inherent in retrospective measures. Furthermore, we include a comparison group of patients who undergo a cancer biopsy but receive a benign result (ie, no cancer diagnosis) and their spouses or partners. Although it is likely that patients with cancer and their spouses or partners will demonstrate greater decrements in QoL than couples in our benign biopsy comparison group, patients who receive a benign biopsy result and their spouses or partners may also experience some impact as a result of the mortality salience related to a “cancer scare.”

### Limitations

Limitations include primary reliance on clinic-based recruitment in 1 academic medical center in a metropolitan city, which constrains generalizability including racial or ethnic representation. Furthermore, we focused exclusively on patients undergoing breast or prostate biopsies, which are 2 of the most common cancers. Future research should focus on other tumor types that have received less attention in QoL studies. We also recommend that future researchers who are interested in interrogating adaptation to adversity among couples also consider including behavioral and neurobiological assessments in addition to self-report measures. This study included a 9-month follow-up period, which was chosen to feasibly complete recruitment and assessments within the 2-year grant period. Furthermore, in order to retain sufficient resources to enroll and follow as many couples who received a malignant biopsy result as possible, we were not able to longitudinally follow all couples who received a benign biopsy result. Therefore, more prospective studies are needed with follow-up schedules beyond the first year after a biopsy result, specifically among couples who receive benign biopsy results. Finally, although it is important to understand how individual and relational functioning prior to biopsy predicts QoL following a diagnosis, we acknowledge that this assessment time point is not a true baseline of functioning as patients are undergoing biopsy as a result of abnormal screening findings and symptomatic presentation (of which they and their spouses or partners are aware).

### Conclusions

A cancer diagnosis is a profound and often very stressful experience for both patients and their partners. Spouses and partners are by far the most common informal cancer caregivers, helping patients manage disease symptoms, treatment side effects, and emotional distress. To our knowledge, the Couples Cope Study is the first study to prospectively examine QoL among both patients and their spouses or partners from the moment prior to a diagnostic biopsy through the year following a malignant or benign diagnosis. The results of this study will address a crucial gap in the literature and contribute to our understanding of how individual well-being and relationship quality contribute to QoL outcomes among couples facing cancer and can inform our future efforts to provide effective interventions and support earlier to couples facing a possible cancer diagnosis.

## Appendix

Multimedia Appendix 1 Baseline and follow-up study assessments.

## Abbreviations

CONSORT: Consolidated Standards of Reporting Trials
EHR: electronic health record
QoL: quality of life

## Data Availability

Data from this study are not publicly available due to ongoing analyses; however, deidentified data from this study will be made available from the corresponding author upon reasonable request (as allowable according to institutional review board standards).
