# Supplementary material for: Quality of Life in Patients and Their Spouses and Cohabitating Partners in the Year Following a Cancer Biopsy (the Couples Cope Study): Protocol for a Prospective Observational Study
Source: JMIR Res Protoc. 2024 Jun 18;13:e52361. doi: 10.2196/52361 (PMC11220434; doi:10.2196/52361)
Supplement: Multimedia Appendix 1 [file resprot_v13i1e52361_app1.docx]

| Domain | Variable | Measure | Time point | Source of data |
| --- | --- | --- | --- | --- |
| **Sociodemographic characteristics** | | | | |
|  | Age | Age in years | Baseline | Self-report |
|  | Sex assigned at birth | Male, female, and intersex | Baseline | Self-report |
|  | Gender identity | Cis man, cis woman, trans man, trans woman, nonbinary, and not listed (please specify) | Baseline | Self-report |
|  | Sexual orientation | Heterosexual (or straight), gay or lesbian, bisexual, not listed (please specify), and prefer not to answer | Baseline | Self-report |
|  | Continued relationship with spouse or partner | Yes, no, and other (please specify) | 1, 6, and 9 months | Self-report |
|  | Type of relationship | Married, living (“cohabitating”) with a romantic partner, and other | Baseline | Self-report |
|  | Relationship length | In years and months | Baseline | Self-report |
|  | Married more than once | Yes, no, and prefer not to answer | Baseline | Self-report |
|  | Number of marriages | Write-in number of marriages | Baseline | Self-report |
|  | Cohabitating history | Yes, no, and prefer not to answer | Baseline | Self-report |
|  | Number of cohabitations | Write-in number of cohabitations | Baseline | Self-report |
|  | Parental status | Yes, no, and prefer not to answer | Baseline | Self-report |
|  | Number of children | Write-in number of children | Baseline | Self-report |
|  | Highest level of education | None, some elementary, completed elementary school, some high school, graduated high school (diploma or General Educational Development [GED]), 2-year technical degree, associate degree, some college, 4-year bachelor’s degree, master’s degree, doctorate degree, and prefer not to answer | Baseline | Self-report |
|  | Race | White, Black, Asian, Native American, multiracial, and prefer not to answer | Baseline | Self-report |
|  | Ethnicity | Hispanic or Latino/a (yes, no, and prefer not to answer) | Baseline | Self-report |
|  | Education | None, some elementary, completed elementary school, some high school, graduated high school, 2-year technical degree, associate degree, some college, 4-year bachelor’s degree, master’s degree, doctorate degree, and prefer not to answer | Baseline | Self-report |
|  | Health insurance | None, no insurance and without coverage; coverage through current or former employer or labor union; coverage through individual plan; coverage through Medicaid or Medicare; other (please specify); and prefer not to answer | Baseline | Self-report |
|  | Household income | Unemployed, retired, less than US $25,000; US $25,000-US $49,000; US $50,000-US $74,999; US $75,000-US $99,999; US $100,000-US $124,999; US $125,000-US $149,000; US $150,000 and greater; and do not know | Baseline | Self-report |
|  | People supported by household income | Write-in number of people being supported by household income | Baseline | Self-report |
| **Medical characteristics** | | | | |
|  | Medical conditions | High blood pressure (hypertension), high cholesterol, arthritis, heart disease (heart attack, angina, and heart failure), diabetes, kidney disease, thyroid problems, autoimmune disease (lupus, Sjogren’s, rheumatoid arthritis, and multiple sclerosis), and other (please specify) | Baseline | Self-report |
|  | Biopsy results | Malignant (cancer diagnosis) or benign (no cancer diagnosis) | 1 month | EHR^a^ |
|  | Cancer stage | 0-IV | 1 month | EHR |
|  | Cancer type | Prostate, breast, and other (please specify) | 1, 6, and 9 months | EHR and self-report (patients with cancer only) |
|  | Diagnosis date | Write-in year and month | 1, 6, and 9 months | EHR and self-report (patients with cancer only) |
|  | Receiving cancer treatment | Yes, no, and unsure | 1, 6, and 9 months | EHR and self-report (patients with cancer only) |
|  | Current cancer treatments | Surgery, radiation, chemotherapy, hormone therapy, immunotherapy, cryotherapy, active surveillance, and other (please specify) | 1, 6, and 9 months | EHR and self-report (patients with cancer only) |
|  | Initiation date of current treatments | Write-in month and year each current cancer treatment endorsed was started | 1, 6, and 9 months | EHR and self-report (patients with cancer only) |
|  | Past cancer treatments | Yes, no, and unsure | 1, 6, and 9 months | Self-report (patients with cancer only) |
|  | Type of past treatment | Surgery, radiation, chemotherapy, hormone therapy, immunotherapy, cryotherapy, active surveillance, and other (please specify) | 1, 6, and 9 months | Self-report (patients with cancer only) |
|  | Completion date of past treatments | Write-in month and year each past cancer treatment endorsed was completed | 1, 6, and 9 months | Self-report (patients with cancer only) |
| **Individual well-being** | | | | |
|  | Gratitude | Gratitude Questionnaire 6-Item Form [28] | Baseline, 1, 6, and 9 months | Self-report and peer-report (follow-ups only) |
|  | Empathy | Interpersonal Reactivity Index [29] | Baseline, 1, 6, and 9 months | Self-report and peer-report (follow-ups only) |
|  | Meaning and purpose | PROMIS Meaning and Purpose [30] | Baseline, 1, 6, and 9 months | Self-report and peer-report (follow-ups only) |
|  | Attachment style | Experiences in Close Relationships questionnaire [31] | Baseline, 1, 6, and 9 months | Self-report |
|  | Relatedness (perceived social support) | Multidimensional scale of Perceived Social Support [32] | Baseline, 1, 6, and 9 months | Self-report |
|  | Emotional support network | Adapted Emotionships items [33] | 1 month | Self-report |
| **Relationship well-being** | | | | |
|  | Relationship well-being | Perceived Relationship Quality Components inventory [34] | Baseline, 1, 6, and 9 months | Self-report |
|  | Social constraints | Social Constraints on Disclosure about Cancer items [35] | Baseline, 1, 6, and 9 months | Self-report |
| **Perceived growth** | | | | |
|  | Retrospective perceived growth | Post-Traumatic Growth Inventory [36] | 9 months | Self-report |
| **Quality of life** | | | | |
|  | Depression | Center for Epidemiologic Studies Depression Scale: Self-Report [37] | Baseline, 1, 6, and 9 months | Self-report |
|  | Positive affect | Modified Differential Emotions Scale [38] | Baseline, 1, 6, and 9 months | Self-report |
|  | Negative affect | Modified Differential Emotions Scale [38] | Baseline, 1, 6, and 9 months | Self-report |
|  | Life satisfaction | Satisfaction with Life Scale [39] | Baseline, 1, 6, and 9 months | Self-report |
|  | Stressful life events | Brief Life Events Checklist [40] | Baseline | Self-report |
|  | Financial toxicity | Comprehensive Score for Financial Toxicity Scale [41] | Baseline, 1, 6, and 9 months | Self-report |
|  | Sexual functioning | PROMIS Sexual Function and Satisfaction Brief Profile [42] | Baseline, 1, 6, and 9 months | Self-report |
| **Cancer-specific measures** | | | | |
|  | Health-related quality of life | Functional Assessment of Cancer Therapy-General [43] plus breast and prostate cancer symptom subscales [44,45] | 1, 6, and 9 months | Self-report (patients with cancer only) |
|  | Intrusive thoughts and avoidance | Impact of Events Scale [46] | 1, 6, and 9 months | Self-report (patients with cancer only) |
|  | Supportive care needs | Supportive Care Needs Survey [47,48] | 1, 6, and 9 months | Self-report (patients with cancer only) |

^a^EHR: electronic health record.
